# Ovarian Torsion Secondary to a Dermoid Cyst in a Child: A Case Report

**DOI:** 10.7759/cureus.86565

**Published:** 2025-06-22

**Authors:** Muhammad Mudasir Saleem, Mishal Pervaiz, Ismail Mazhar, Amna Waseem, Mahnoor Yawar Irfan Khan, Maham Sultan, Maaz Safdar Khan, Saeed Ur Rehman

**Affiliations:** 1 Pediatric and General Surgery, Combined Military Hospital (CMH) Lahore, Lahore, PAK; 2 Anesthesiology, Punjab Rangers Teaching Hospital, Lahore, PAK; 3 Pediatric Surgery, Combined Military Hospital (CMH) Lahore Medical College and Institute of Dentistry, Lahore, PAK; 4 Anatomy, Combined Military Hospital (CMH) Lahore Medical College and Institute of Dentistry, Lahore, PAK; 5 Pediatric Surgery, Loralai Medical College, Loralai, PAK

**Keywords:** child, cyst, dermoid, ovary, torsion

## Abstract

A four-year-old girl presented with a two-day history of progressively worsening right lower abdominal pain, accompanied by anorexia and nausea. The initial assessment revealed lower abdominal tenderness with guarding and rebound tenderness, raising concern for an acute surgical abdomen. Ultrasonography demonstrated mild ascites and an enlarged left ovary with absent Doppler flow, suggestive of torsion. Emergent laparoscopic exploration revealed a 6×7 cm necrotic left adnexal mass with complete torsion of the ovary and fallopian tube. Detorsion was attempted, but the ovary remained nonviable, necessitating a left salpingo-oophorectomy. Gross examination revealed hair within the mass, and histopathology confirmed an infarcted ovarian dermoid cyst. The child had an uneventful recovery and was well at both the one- and three-month follow-ups. This case underscores the importance of the early recognition and prompt surgical management of pediatric ovarian torsion to prevent irreversible adnexal damage and preserve future fertility whenever possible.

## Introduction

Ovarian torsion occurs due to the twisting of the ovary around its vascular pedicle, which is formed by the infundibulopelvic and tubo-ovarian ligaments. It has an estimated incidence of approximately five per 100,000 women, primarily affecting those between 1 and 20 years of age [1]. Several risk factors contribute to ovarian torsion, with the presence of an ovarian mass being the most significant, accounting for up to 80% of cases in the pediatric population [2].

Mature cystic teratoma, also known as a dermoid cyst, is the most common ovarian mass in children [3]. Dermoid cysts are benign tumors arising from ovarian totipotent cells, which have the potential to develop into well-differentiated tissues derived from all three germ layers: the ectoderm, mesoderm, and endoderm [4]. Clinically, ovarian torsion presents with severe lower abdominal pain, often accompanied by vomiting. The pain typically worsens over time as ischemia progresses [5]. Doppler ultrasonography is a reliable diagnostic tool revealing reduced or absent blood flow to the affected ovary [6].

Early diagnosis and prompt intervention are crucial to prevent the need for oophorectomy. Here, we present a case of ovarian torsion in a four-year-old girl with a dermoid cyst. She presented with features of an acute abdomen and was diagnosed with ovarian torsion on Doppler ultrasound. Laparoscopic detorsion was attempted; however, ovarian preservation was not possible due to the prolonged duration of symptoms caused by delayed presentation.

## Case presentation

A four-year-old girl, weighing 17 kg, presented to the pediatric surgery emergency with a history of sudden-onset, progressively increasing, right-sided lower abdominal pain for two days. The pain was associated with anorexia and nausea. Initially, her parents consulted a general practitioner who prescribed oral analgesics, resulting in temporary relief of her symptoms. As the pain continued to worsen in severity, her parents sought further evaluation at the pediatric surgery emergency department. The parents deny any history of abdominal trauma.

On initial assessment, her vital signs were within normal limits. Physical examination revealed a non-distended abdomen with tenderness across the lower abdomen, accompanied by guarding and rebound tenderness. No palpable mass was detected, and bowel sounds were audible. Both hernial orifices were intact.

Abdominal ultrasonography showed mild abdominopelvic ascites and an enlarged left ovary with no detectable blood flow on Doppler ultrasound, highly suggestive of ovarian torsion. The contralateral ovary appeared normal. Baseline investigations were ordered, and an emergent laparoscopic exploration was planned after explaining the details to her parents. Informed written consent was obtained.

**Figure 1:**
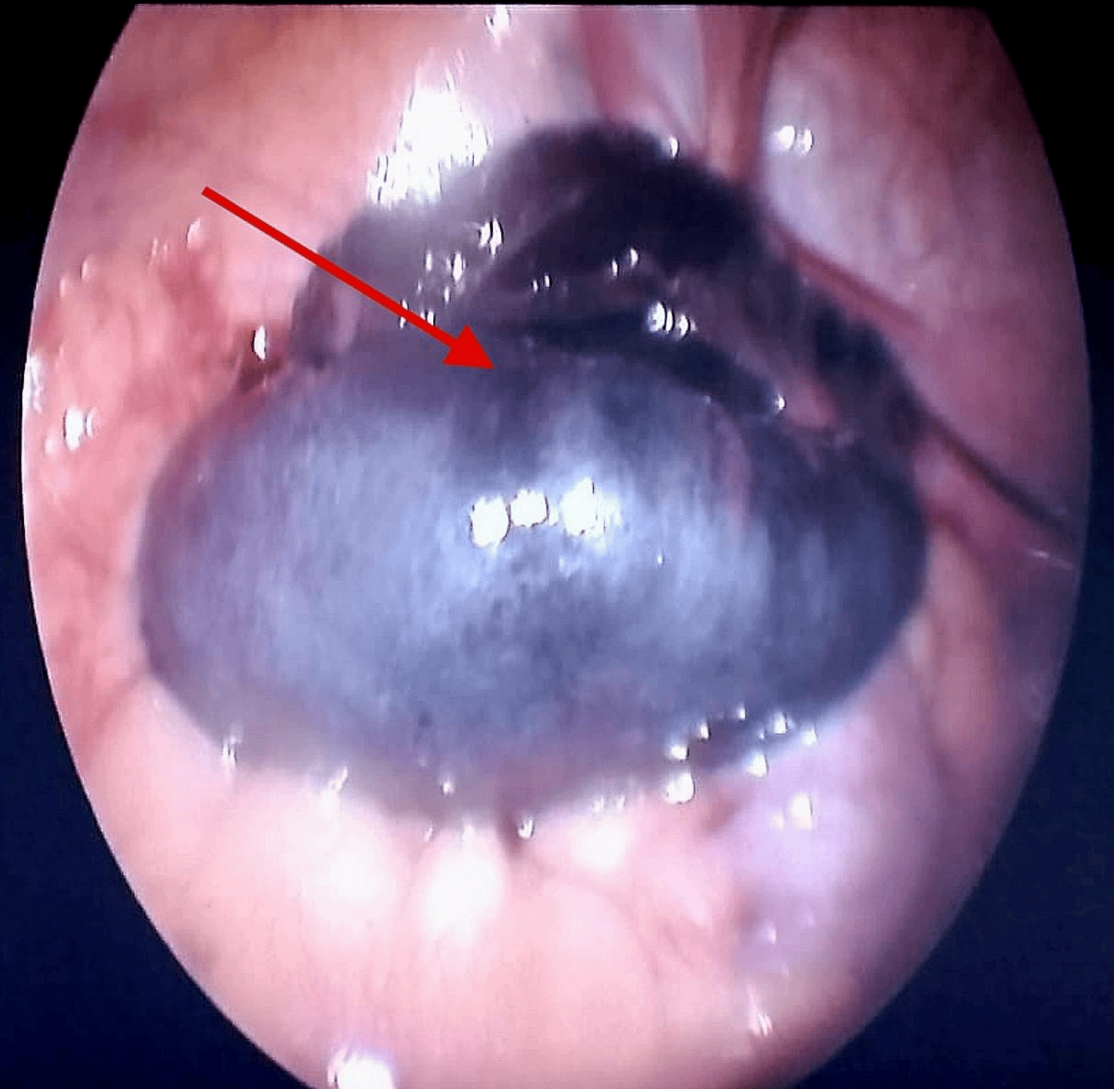
Laparoscopic image showing a necrotic, bluish-black, twisted left adnexal mass (arrow) consistent with ovarian torsion. The ovary appears enlarged and devitalized due to compromised blood supply.

During surgery, pneumoperitoneum was established via a 5 mm umbilical port. Inspection revealed reactionary fluid in the pelvis and a 6×7 cm left adnexal mass with the complete torsion of the ovary and fallopian tube (Figure 1).

**Figure 2:**
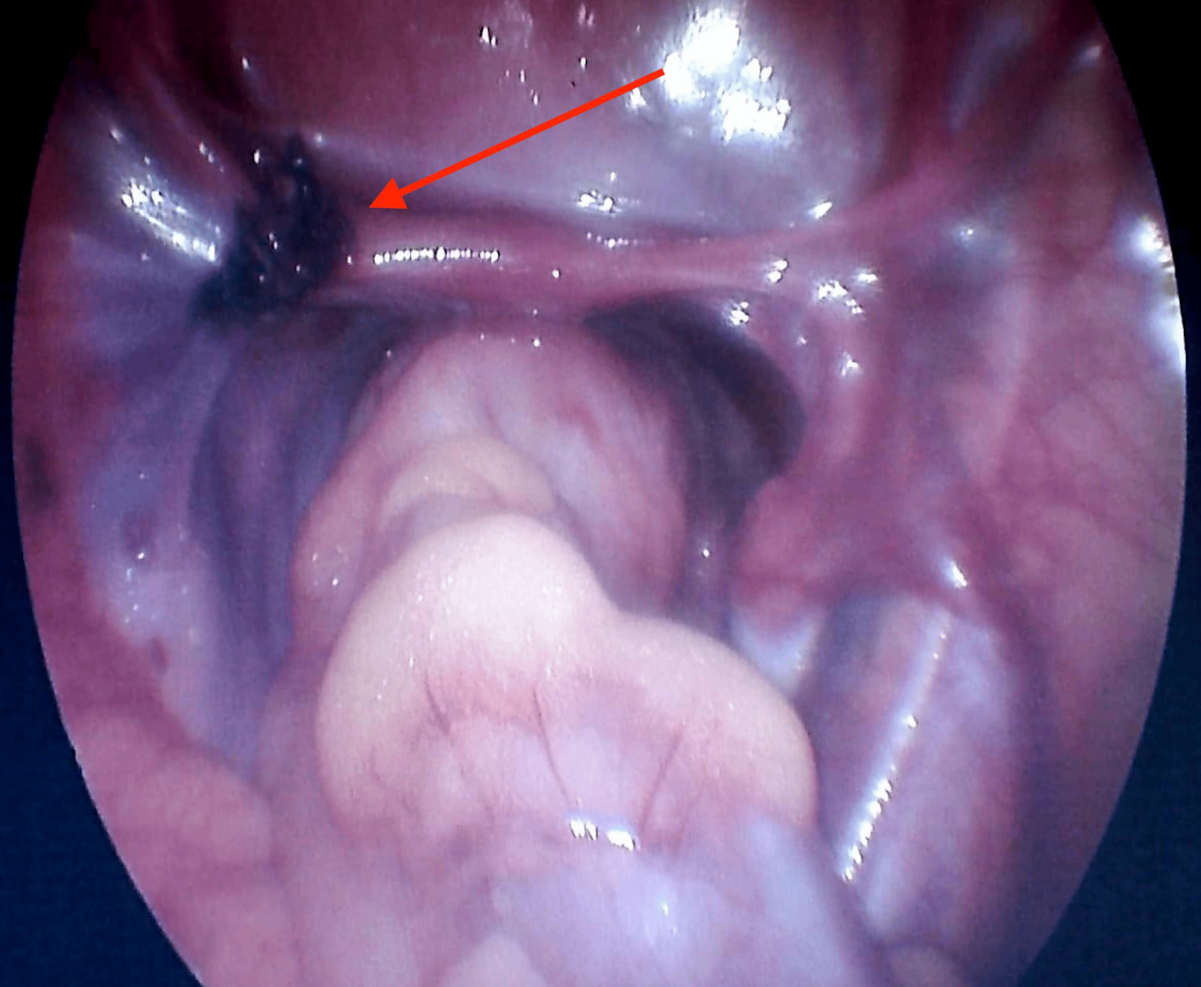
Intraoperative view following left salpingo-oophorectomy. The arrow indicates the site of ligation at the base of the removed adnexal structures. Normal-appearing bowel and peritoneum are also seen.

Two additional 5 mm working ports were placed in the right and left upper abdomen, followed by the detorsion of the twisted adnexal structures. A complex, hard, necrotic mass was noted involving the entire left ovary with no improvement in color after detorsion. Consequently, a left salpingo-oophorectomy was performed with the base ligated using Vicryl 2-0 (Ethicon, Inc., Raritan, NJ) (Figure 2). The right ovary and fallopian tube appeared normal.

**Figure 3:**
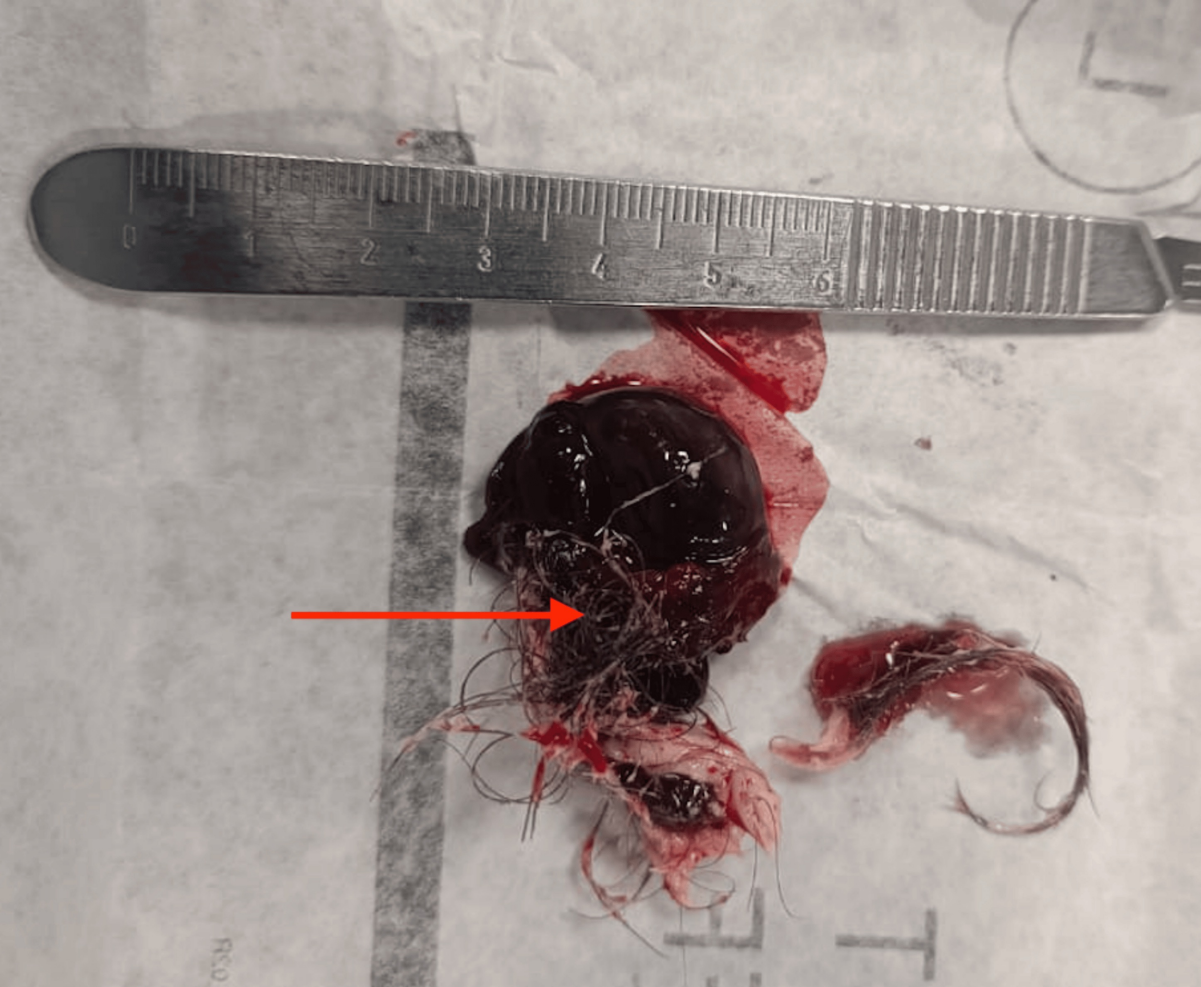
Gross specimen retrieved postoperatively, showing a dermoid cyst. The arrow points to a tuft of hair, a classical component of mature cystic teratomas.

Specimen retrieval was done in a piecemeal fashion using an endo bag, revealing a tuft of hair (Figure 3).

**Figure 4:**
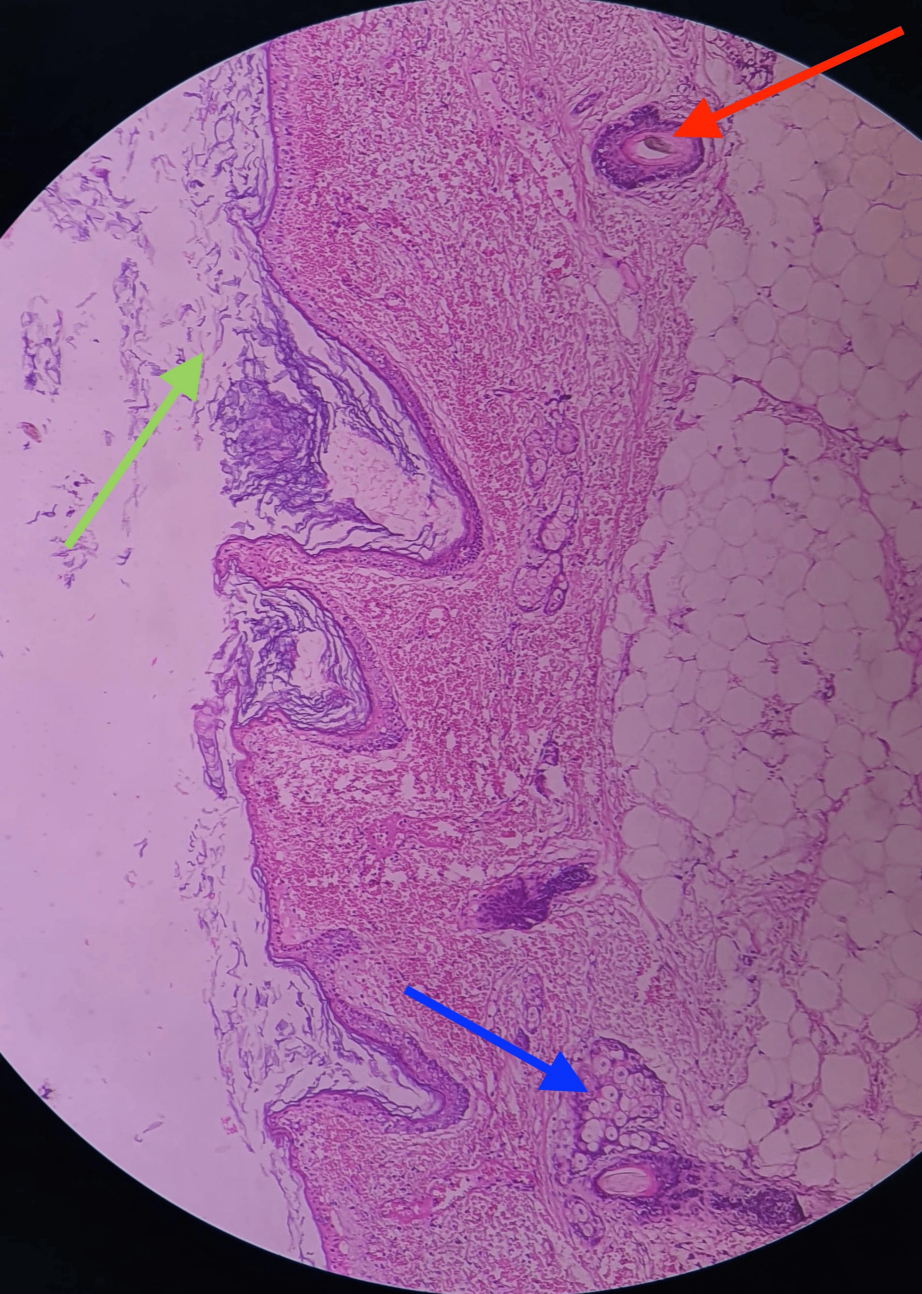
Hematoxylin and eosin (H&E) stain, original magnification: ×400. The section demonstrates features of a dermoid cyst with infarcted ovarian tissue. Green arrow, keratin flakes; red arrow, hair follicle; and blue arrow, sebaceous glands.

Histopathological analysis confirmed infarcted ovarian tissue with hemorrhage and a cyst lined by stratified squamous epithelium containing hair follicles, sebaceous glands, occasional sweat glands, and keratin flakes, findings consistent with an ovarian dermoid cyst complicated by torsion (Figure 4).

Postoperatively, the child had an uneventful recovery and was discharged on the third postoperative day. Follow-up at one and three months showed a thriving child with no complaints.

## Discussion

Ovarian torsion is a rare but critical gynecological emergency, particularly in pediatric patients, requiring prompt diagnosis and surgical intervention to preserve ovarian function. Ovarian torsion more commonly occurs on the right side, accounting for approximately 70% of cases. This predominance is partly attributed to the longer utero-ovarian ligament on the right. In contrast, the left side offers less anatomical space due to the presence of the sigmoid colon, which further influences the right-sided occurrence of torsion [7]. In our case however, the patient was diagnosed with ovarian torsion on the left side.

The torsion of ovaries occurs due to the twisting of the ovary and its vascular pedicle, leading to impaired venous and lymphatic drainage, congestion, and eventual arterial compromise [8]. The incidence of pediatric ovarian torsion is estimated to be 4.9 cases per 100,000 women per year, with an increased risk in the presence of an ovarian mass [9]. In our case, a four-year-old girl presented with right lower abdominal pain, which was initially mismanaged with analgesics, leading to a delayed presentation and ultimately the loss of the ovary.

Mature cystic teratomas (dermoid cysts) are the most common ovarian tumors in children, representing up to 50% of pediatric ovarian neoplasms [10]. These benign germ cell tumors arise from totipotent cells and contain tissues derived from all three germ layers. Their presence increases ovarian weight and alters normal adnexal anatomy, predisposing the ovary to torsion [11].

Mature dermoid cysts in the pediatric population are relatively rare and challenging to diagnose due to various symptoms and their tendency to mimic other illnesses. One example is a rare case involving a 31-month-old asymptomatic girl who was evaluated in the emergency department following the incidental discovery of a suprapubic mass. Subsequent imaging revealed characteristic features of a dermoid cyst [12]. Similarly, although CA 19-9 is not typically associated with benign ovarian teratomas, a rare case reported a significantly large dermoid cyst accompanied by elevated CA 19-9 levels. These findings suggest a possible, though not clearly defined, association between the tumor and biomarker elevation [13].

Studies indicate that adnexal pathology accounts for 51%-84% of pediatric adnexal torsion cases, with cystic teratomas or dermoid cysts responsible for 31% and follicular or hemorrhagic ovarian cysts contributing 23%-33% [14]. This aligns with our case, where a 6×7 cm dermoid cyst resulted in the complete torsion of the left ovary.

The clinical presentation of ovarian torsion is often nonspecific, mimicking other causes of acute abdomen, such as appendicitis or gastroenteritis [15]. The classic symptoms include lower abdominal pain, nausea, and vomiting, all of which were present in our patient. However, due to the intermittent nature of torsion and detorsion, symptoms may initially be mild, leading to a delay in seeking appropriate medical care [16]. In our case, the child's parents first consulted a general practitioner who prescribed oral analgesics without advising any workup, allowing ischemia to progress unchecked. This highlights the importance of early recognition and referral, keeping a high index of suspicion for ovarian torsion.

Doppler ultrasonography is the primary imaging modality for diagnosing ovarian torsion, with findings such as an enlarged ovary with reduced or absent blood flow [17]. However, normal Doppler flow does not exclude torsion as arterial supply may be preserved in the early stages [18]. In our case, the ultrasound showed mild abdominopelvic ascites and an enlarged left ovary with absent Doppler flow, confirming the diagnosis.

Laparoscopic management is the preferred surgical approach, offering both diagnostic and therapeutic benefits. Detorsion is the first-line treatment, as studies have shown that even necrotic-appearing ovaries may regain function [19]. However, in our case, the ovary appeared completely necrotic, and despite an attempted detorsion, no color improvement was observed, necessitating a left salpingo-oophorectomy. This aligns with findings by Oelsner et al., who noted that prolonged ischemia often results in irreversible ovarian necrosis, requiring oophorectomy [20].

The loss of an ovary at a young age carries significant long-term consequences, including reduced ovarian reserve and potential hormonal imbalances [21]. Additionally, the psychological impact on both the child and parents is profound, making timely intervention crucial. Current recommendations emphasize ovarian preservation whenever possible, particularly in pediatric patients [22]. Ovarian cystectomy following detorsion is the treatment of choice, and oophoropexy may be considered to reduce the risk of recurrence [23]. Unfortunately, in our case, the delayed presentation led to a point of no return for ovarian salvage. Histopathology confirmed the presence of a dermoid cyst with superimposed torsion, which is consistent with previously reported cases [24].

## Conclusions

Ovarian torsion is a time-sensitive surgical emergency requiring a high index of suspicion, especially in pediatric patients with ovarian masses. Early Doppler ultrasound evaluation and laparoscopic intervention are crucial in optimizing ovarian salvage rates. Delayed diagnosis, as seen in our case, may result in irreversible ischemia necessitating oophorectomy. Awareness and timely intervention can significantly reduce morbidity and long-term consequences in affected children.
